# Knowledge and Compliance among Contact Lens Wearers Living in Kuala Lumpur, Malaysia: A Cross-Sectional Study

**DOI:** 10.21315/mjms2022.29.5.13

**Published:** 2022-10-28

**Authors:** Bashirah Ishak, Amalina Nur Amiera Azizan, Vanitha Mariappan

**Affiliations:** 1 Program of Optometry and Vision Science, Research Centre for Community Research, Faculty of Health Sciences, Universiti Kebangsaan Malaysia, Kuala Lumpur, Malaysia; 2 Center of Toxicology and Risk Health, Faculty of Health Sciences, Universiti Kebangsaan Malaysia, Kuala Lumpur, Malaysia

**Keywords:** contact lens wearers, compliance, knowledge on contact lens, good practice

## Abstract

**Background:**

Many factors determine the success of wearing contact lens, including knowledge and compliance towards lens care. This study aimed to evaluate the level of knowledge and compliance between two groups of adult contact lens wearers in Kuala Lumpur, Malaysia.

**Methods:**

A total of 60 participants aged 18–30 years old volunteered to participate in this study (30 participants prescribed contact lenses at the Universiti Kebangsaan Malaysia [UKM] optometry clinic and 30 participants fitted at private practices). Participants were interviewed using a structured questionnaire consisting of nine questions on basic knowledge related to lens wear and 13 questions on compliance, categorised into questions on the cleaning process, disinfection, accessories care and replacement schedule.

**Results:**

All participants wore disposable contact lens 53.3% (*n* = 32) participants preferred using monthly disposable lenses. Both groups had the same level of knowledge, except knowledge of the effects of makeup (*P* < 0.000) and duration of the solution used for cleaning (*P* < 0.010), showing lack of knowledge on contact lenses among participants in private practices group. There were no significant differences between the groups in terms of the level of compliance with the cleaning process (*P* = 0.830), disinfection (*P* = 0.725), accessories care (*P* = 0.865) and replacement schedule (*P* = 0.699).

**Conclusion:**

Participants from UKM optometry clinic had better knowledge on wearing contact lens; moreover, both groups had good compliance towards lens care. Contact lens practitioners should provide all the necessary information to contact lens wearers so that they are equipped to handle contact lenses correctly, which would minimise the risk of eye complications.

## Introduction

In the health-care sector, non-compliance to follow the instructions of prescribed medical treatments can cause a substantial upsurge of expenses and health indisposition, eventually leading to escalation of interventional treatments over time (1). Wearing contact lens is a trending fashion among the younger generation, especially among students and younger working adults. Eyeglasses or spectacles are the simplest, cheapest and most frequently used tools for correcting refractive errors. Moreover, no particular compliance needs to be followed on a daily basis while using them.

Contact lens is an optical device that can be safely used for correcting refractive error and for cosmetic or therapeutic purposes (2). Regarding therapeutic purposes, contact lens protects and cures the irregular surface of the cornea (3) due to ocular diseases such as keratoconus and scarring on the cornea. Regarding cosmetics purposes, contact lens alters the colour of the eyes to make them look more attractive (3) and increase their aesthetic value.

In addition, knowledge on handling and wearing contact lens correctly can help to prevent complications associated with non-compliance with the instructions provided (4). Good knowledge from the beginning can help avoid inappropriate use and ensure compliance among contact lens wearers (5). The level of knowledge can be determined by measuring the perceptions of the wearer based on their knowledge about contact lens (4). According to Tajunisah et al. (2) all contact lens wearers should have sufficient knowledge on good maintenance process and contact lens complications such as microbial keratitis.

Ashbool et al. (6) defined compliance as the extent to which a patient’s behaviour coincides with the clinical prescription by medical practitioners. An individual with good compliance is the one who washes his/her hands before handling the contact lens and practices the good cleaning process, proposed by Food and Drug Administration (FDA). He/she also does not overwear the contact lens for an extended time that is not admitted by FDA, the cleaning solution and lens cases are not contaminated by bacteria (7). The lens maintenance procedures should be recommended by certified practitioners such as optometrists while prescribing the lens and the wearers must observe and follow the instructions accordingly. Although aspects that lead to recalcitrant behaviour are unclear, the major contributing factors appear to be financial constrain, elongated required time and intricacy of treatment or schedule. In order to improve amenability, the factual proportion of compliance should be resolute. However, the development of actual levels of compliance is enervating with faults since there is no reliable test to quantify or measure compliance.

Bui et al. (8) reported that two-thirds of 100 participants admitted that they were not compliant while handling the contact lens. The remaining participants admitted that they were compliant and used a good and safe cleaning process of contact lens. Most of the participants wore contact lens when swimming, did not top up the solution, used tap water and failed to replace the lens case with a new one. Some factors, such as economic problems, complicated instructions by practitioners, complex and long time needed for the maintenance process, contribute to the non-compliance behaviours (8).

In this study, two groups of participants were included; one group was fitted at Universiti Kebangsaan Malaysia (UKM) optometry clinic and the other group was fitted at private practices. UKM optometry clinic conducts a full contact lens fitting examination before prescribing the lens to the patients. Private practices are clinics or optical shops that are also allowed to prescribe the lenses to the patients. However, it is unclear whether private practices conduct a full contact lens fitting examination or just prescribed the lenses over the counter. Therefore, we presumed that patients in both groups received different extent of contact lens fitting consultation. This study was conducted to evaluate the level of knowledge and compliance among these two groups of adult contact lens wearers in Kuala Lumpur, Malaysia.

## Methods

### Instruments

This study was conducted at the Optometry Primary Eye Care Clinic, UKM, Kuala Lumpur. Informed consent was obtained from all participants and the data kept private. The purposive sampling method was used in this study. The sample size was estimated using the sample size formula proposed by Krejcie and Morgan (9), considering an additional 10% potential dropout rate. Although a minimum sample size of 82 was required, only 60 participants who completed the questionnaires were included for analysis due to time constraints. Therefore, 60 soft contact lens wearers (30 participants from UKM Optometry Clinic and 30 participants from private practices) were included. A cross-sectional study was conducted using an adapted questionnaire (available in both English and Bahasa Malaysia) consisting of nine questions related to basic knowledge on lens regime, symptoms experienced while wearing lens, side effects of makeup, and overwear syndrome based on knowledge, attitude and practice by Janti et al. (10). Additionally, 13 questions on compliance were categorised into four sections; lens cleaning procedures, disinfection, accessories care and replacement schedule based on previously validated questionnaire by Bui et al. (8). The inclusion criteria were Malaysian participants aged 18 years old–30 years old, conventional or disposable soft contact lens wearers (hydrogel/silicone hydrogel material) for at least 3 months with good general and ocular health, visual acuity of 6/6 after correction with spherical correction between −1.00 and −6.00 DS and astigmatism of less than −1.00 DC. Participants who did not meet the above criteria were excluded.

## Data Collection Process

The data were collected from January to May 2018. Potential participants were identified according to the inclusion and exclusion criteria. Then, participants were given the information sheet and written consent form to be completed if the respondents agreed to participate in the study. Participants were interviewed using a structured questionnaire before leaving the clinic.

## Data Analysis

Data were collected, tabulated and analysed using SPSS software version 23.0. Descriptive analyses were performed to outline the knowledge and compliance related to contact lens usage among participant from the UKM Optometry Clinic and private practices. A normality test (Shapiro-Wilk test) was performed to determine data distribution prior to statistical analysis of the data. Inferential analyses were performed at the significant level of *P* < 0.050 using an unpaired *t*-test.

## Results

### Demographic Characteristics

The mean age of the participants was 21.8 (SD = 1.9) years old. All the recruited participants were women and who wore disposable soft contact lenses. Thirty-two (53.3%) participants wore monthly disposable lenses, 12 (20.0%) participants wore biweekly disposable lenses and 16 (26.7%) participants wore daily disposable lenses. Most participants were Malay (61.7%), followed by Chinese (33.3%), the remaining were Indian or of other ethnicity. Table 1 shows the distribution of participants according to their preferred disposable lenses and races.

### Basic Knowledge among Contact Lens Wearers

Most participants (*n* = 59, 98.0%) wore contact lenses to correct their refractive errors and look good without eyeglasses. Only one participant solely wore contact lenses for cosmetic purposes. Table 2 shows the percentage of correct responses regarding basic knowledge on lens wear between the groups. All participants (100%) knew that they had to remove contact lens before going to sleep. Most participants had some basic knowledge regarding contact lens usage; however, both groups generally had low knowledge on the side effects of the eye makeup (13.0%–57.0%). The UKM Optometry Clinic group showed higher percentage in the five items of basic knowledge related to contact lens usage, while the private practices group showed a higher percentage only in one item. In the remaining three items, both groups showed 100%. Overall, the UKM Optometry Clinic group had a higher (82.6%) basic knowledge on contact lens use than the private practice group (68.5%).

### Practice of Hygiene and Care of Contact Lens (Compliance)

In terms of compliance, the questionnaire was divided into four sections based on the lens cleaning process, lens care system, accessories care and lens replacement schedule. Participants preferred to use multipurpose solutions for their lens care regime because of the all-in-one care system. Overall, both groups had good compliance with lens care. Both groups showed low compliance with the lens disinfecting system (67%–70%). Table 3 shows the percentage of correct responses regarding compliance with lens use in both groups. In general, the UKM optometry clinic and private practices groups showed a high compliance with lens use (87.5% and 89.2%, respectively).

### Comparison between Basic Knowledge and Level of Compliance between the Groups

Participants in both groups showed a low percentage of knowledge regarding the side effects of eye makeup on contact lens wear. There was a significant difference in this knowledge between the groups (*P* < 0.000). Only 36.7% (*n* = 11) participants in the private practices group had knowledge on the expiration of lens solution, while (70.0%, *n* = 21) participants into the UKM Optometry Clinic group had this knowledge. There was a significant difference in this knowledge between the groups (*P* = 0.010). These findings indicated that contact lens wearers from UKM optometry clinic had better knowledge related to lens wear then private practices group.

However, there were no significant differences between the groups in terms of the level of compliance with the lens cleaning process (*P* = 0.830), lens care system (*P* = 0.725), accessories care (*P* = 0.865) and lens replacement schedule (*P* = 0.699) indicating that both groups had good compliance towards lens care.

## Discussion

This study was aimed to determine the association between apparent compliance with knowledge of contact lens use related to complications and awareness. Currently, in Malaysia, limited studies are available on the knowledge and prevalence of contact lens use, despite the fact that most students and young working adults wear contact lenses. All our study participants were women, consistent with the findings if a previous study, showing that most contact lens wears are women and that the main reason of using lenses was cosmetic, particularly to enhance the beauty and pleasant appearance compared to wearing spectacles (10).

In our study, both groups showed a lack of knowledge on the effects of eye makeup on contact lens, the expiration of lens solution and eye infection related to contact lens use. Similarly, Janti et al. (10) reported that many medical college students in Tamil Nadu, India are unaware of the effects of eye makeup and eye infections related to contact lens use. Additionally, they noticed that some students were aware of the cleaning material and used lens solution; however, a small number of students used water to substitute lens solution when it was not available.

Use of eye makeup such as mascara on eyelashes can induce microparticles, which migrate and cause tear film instability (11). Microparticles from various types of cosmetic products can adhere to the corneal epithelium layer and conjunctiva (12) and can cause vision disturbance (13), contact dermatitis and loss of lashes (14).

Wu et al. (15) reported that different types of contact lens solutions might have different formulations and efficacy to kill bacteria. Each product becomes more efficient if the users follow the instructions provided by the manufacturers. The users must always read the instructions so that they know how to use it and the duration needed to soak the lens or be aware of the products expiry dates.

In our study, almost all participants followed good and hygienic lens cleaning processes. According to Jumaa (16), good handwashing is essential and important to control and kill the bacteria, preventing them to adhering on the contact lens surfaces and reducing eye infections. In addition, Leivens et al. (17) have suggested that it is crucial to give more time to handwashing with extra amount of soap, especially in the area that is exposed to dirt or bacteria (under nails). Nevertheless, cleaning the contact lens is also compulsory to get rid of the debris and adhered bacteria on the lens (18).

Our data showed that 95% participants disinfected and soaked their lenses using the correct technique. A proper way of disinfecting the lenses can make its use safer (19). In general, optometrists have suggested cleaning the lens case (including the lid), using lens solution daily and drying the lens case before use. Moreover, lens cases should be replaced every 90 days to avoid any contamination (5). In a study by Chang et al. (20), approximately 30% participants revealed that they clean their lens case on daily basis, but mainly with tap water, which may cause eye infections.

Ishak et al. (21) reported that 63.4% contact lens wearers showed good compliance towards lens care. In their study, they included students from UKM who wore soft disposable contact lenses for at least 6 months. They used the same questionnaire as that used in this study to ascertain the compliance level among their participants. Our study showed higher compliance level (87.5%–89.2%) than that reported Ishak et al. (21). The dissimilar criteria of minimum period of lens wear might led to different finding.

## Conclusion

We concluded that both groups had the same level of knowledge except knowledge of the effect of eye makeup on the eyes and knowledge on the expiration of lens solution. Both groups showed good compliance with cleaning, disinfection, maintaining lens accessories and replacement schedules. However, there is a need for contact lens practitioners to educate the consumers on contact lens care and possible complications so that they have good knowledge on how to handle contact lenses appropriately.

## Figures and Tables

**Table 1:** Distribution of type of disposable contact lenses and races in two groups of participants

		UKM optometry clinic group (*n* = 30) *n* (%)	Private practices group (*n* = 30) *n* (%)
Type of disposable contact lenses	Dailies	11 (36.7%)	5 (16.7%)
	Biweekly	8 (26.6%)	4 (13.3%)
	Monthly	11 (36.7%)	21 (70.0%)
Races	Malay	18 (60.0%)	19 (63.3%)
	Chinese	10 (33.3%)	10 (33.3%)
	Indian	1 (3.3%)	1 (3.3%)
	Others	1 (3.3%)	0 (0%)

**Table 2:** Response on basic knowledge of contact lens wear in two groups of participants

Question/Statement	UKM optometry clinic group (*n* = 30) *n* (% subjects with positive response)	Private practices group (*n* = 30) *n* (% subjects with positive response)	*P*-value (^*^ sig *P* < 0.050)
Solution to clean, rinse, disinfect and store the lenses	28 (93.3%)	30 (100%)	0.154
Remove contact lens before going to sleep	30 (100%)	30 (100%)	1.000
Wash hand before handling lens	29 (96.7%)	29 (96.7%)	1.000
Clean contact lens immediately after remove from eye	29 (96.7%)	29 (96.7%)	1.000
Have knowledge of over wear syndrome	24 (80.0%)	17 (56.7%)	0.054
Have knowledge about side effects of eye makeup	17 (56.7%)	4 (13.3%)	< 0.000^*^
Have knowledge about the expiration of lens solution	21 (70.0%)	11 (36.7%)	0.010^*^
Have knowledge about eye infections	21 (70.0%)	16 (53.3%)	0.188
Have knowledge of contact lens versus swimming	24 (80.0%)	19 (63.3%)	0.155

**Table 3:** Response on compliance in two groups of participants

Section	UKM optometry clinic group (*n* = 30) *n* (% subjects with correct response)	Private practices group (*n* = 30) *n* (% subjects with correct response)	*P*-value (^*^ sig *P* < 0.050)
Lens cleaning process	28 (93.3%)	30 (100%)	0.830
Lens care system	20 (66.7%)	21 (70.0%)	0.725
Accessories care	29 (96.7%)	29 (96.7%)	0.865
Lens replacement schedule	28 (93.3%)	27 (90.0%)	0.699
